# Application of the Marshall-Olkin-Weibull logarithmic distribution to complete and censored data

**DOI:** 10.1016/j.heliyon.2024.e34170

**Published:** 2024-07-09

**Authors:** Regent Retrospect Musekwa, Lesego Gabaitiri, Boikanyo Makubate

**Affiliations:** Department of Mathematics and Statistical Sciences, Botswana International University of Science and Technology, Palapye, Botswana

**Keywords:** 62E10, 60E30, Marshall-Olkin-G distribution, Power series distribution, Weibull distribution, Logarithmic distribution, Censored data

## Abstract

In contemporary statistical research, there has been a notable surge of interest surrounding a suggested extension of the Marshall-Olkin-G distributions. The present extension exhibits a higher degree of flexibility in comparison to its parent distributions. In a similar manner, we present in this context an expansion of the Marshall-Olkin-G distributions proposed by statistical scholars. This study utilizes a specific variant of the extension known as the Marshall-Olkin-Weibull Logarithmic model, which is applied to both complete and censored data sets. It is evident that the aforementioned model has strong competitiveness in accurately characterizing both complete and censored observations in lifetime reliability issues, when compared to other comparative models discussed in this research work.

## 1 Introduction

The statistical literature has advocated the addition of at least one parameter to existing distributions or families of distributions, resulting in the creation of several new classes of distributions. Among them, the Marshall-Olkin distribution [1] has garnered attention from academics in the field of statistics. As such [2] developed the Type II Exponentiated Half Logistic-Marshall-Olkin-G family of distributions, and [3] developed the Marshall-Olkin Topp-Leone Half-Logistic-G family of distributions. More research on Marshall-Olkin and on Power Series distribution has been done (see [4], [5], [6], [7], [8], [9] and [10] for details). There is also a recently proposed new model called the Marshall-Olkin-G power series (MO-GPS) [11]. The MO-GPS family was developed by the truncation of the Marshall-Olkin family and the power series to improve flexibility in modeling real life data. We were motivated by the versatility of the MO-GPS and we had to study its behavior when fitting complete and censored data.

Censored data occurs when a research study concludes prior to the occurrence of the event of interest for all subjects under investigation. In this study, data acquired from life-testing experiments involving n samples was utilized. The samples were monitored until the point of failure was reached. The samples included in the reliability experiment may encompass a range of electrical or mechanical components, systems, persons, or even computer chips. Patients, volunteers, or individuals may also be recruited for medication or clinical trials. In the majority of instances, such experiments are concluded prior to the occurrence of failure in all the samples. The aforementioned situation can also be observed in pharmacological or clinical studies, where the individuals who were initially enrolled as subjects may have become lost to follow-up, voluntarily discontinued their participation, withdrew from the study, or the study itself may have been terminated owing to unanticipated circumstances, economic difficulties, or the completion of the designated time frame. Additionally, samples have the potential to inadvertently break or malfunction during an industrial experiment, resulting in what is commonly referred to as a ‘‘failure’’. In general, data collected in these or comparable conditions is referred to as censored data.

Literature has shown different censoring types [12], when a study is conducted over a set length of time that may end before all of the units have failed, thus type I censoring. Every person has a set censoring time, which is the interval between the study's start date and completion. As a result, an individual's entire failure time can only be determined if the amount is the same as or less than the censoring time otherwise, only a lower bound of the person's lifetime is available. Type II censoring occurs when an experiment with a specific number of subjects or objects is halted when a predetermined number are found to have failed; the remaining subjects are then right-censored. In contrast to type I censoring, which involves a set research time and an entirely random number of censored observations, type II double censoring involves missing a fixed number of observations at both ends of a sample size.

Following the notation [11], the cumulative density function (cdf) and the probability density function (pdf) of the MO-GPS family of distributions is as follows;

(1) $$F_{MO-GPS}(x;\delta ,\theta ,\zeta )=1-\frac{C[\frac{\theta \delta \overset{¯}{G}(x;\zeta )}{1-\overset{¯}{\delta }\overset{¯}{G}(x;\zeta )}]}{C(\theta )},$$

and

(2) $$f_{MO-GPS}(x;\delta ,\theta ,\zeta )=\frac{\theta \delta g(x;\zeta )C^{\prime }[\frac{\theta \delta \overset{¯}{G}(x;\zeta )}{1-\overset{¯}{\delta }\overset{¯}{G}(x;\zeta )}]{\left[1-\overset{¯}{\delta }\overset{¯}{G}(x;\zeta )\right]}^{-2}}{C(\theta )},$$

respectively, for δ,θ>0, $\overset{¯}{\delta }=1-\delta$, *ζ* is parameter vector and $C^{\prime }(\theta )$ is the first derivative of C(θ), that is

$$C^{\prime }(\theta )=\sum_{n=0}^{\infty }(n+1)a_{n+1}\theta^{n}.$$

In this study we are focusing on the special case of the MO-GPS called the Marshall-Olkin Weibull Logarithmic (MO-WL) distribution. The details of the MO-GPS and all its statistical properties have been reported earlier (see [11] for more details). Using equation (1) and (2) and also following the notation [11] the cdf and pdf of the MO-WL model are given by

(3) $$F_{MO-WL}(x;\delta ,\theta ,a,b)=1-\frac{\log[1-\frac{\theta \delta e^{-bx^{a}}}{1-\overset{¯}{\delta }e^{-bx^{a}}}]}{\log(1-\theta )},$$

and

(4) $$f_{MO-WL}(x;\delta ,\theta ,a,b)=\frac{\theta \delta abx^{a-1}e^{-bx^{a}}{\left[1-\frac{\theta \delta e^{-bx^{a}}}{1-\overset{¯}{\delta }e^{-bx^{a}}}\right]}^{-1}}{{\left[1-\overset{¯}{\delta }e^{-bx^{a}}\right]}^{2}(-\log(1-\theta ))},$$

respectively, for x,δ,a,b>0, 0<θ<1 and $\overset{¯}{\delta }=1-\delta$. This distribution takes *δ* as a tilt parameter, *a* and *b* as shape parameters and, *θ* as a scale/tilt parameter. The corresponding hazard rate function (hrf) is derived from equation (3) and (4) is given by

(5) $$h_{MO-WL}(x)=\frac{\theta \delta abx^{a-1}e^{-bx^{a}}{\left[1-\frac{\theta \delta e^{-bx^{a}}}{1-\overset{¯}{\delta }e^{-bx^{a}}}\right]}^{-1}}{{\left(1-\overset{¯}{\delta }e^{-bx^{a}}\right)}^{2}\left(-\log[1-\frac{\theta \delta e^{-bx^{a}}}{1-\overset{¯}{\delta }e^{-bx^{a}}}]\right)}.$$

**Figure 1:**
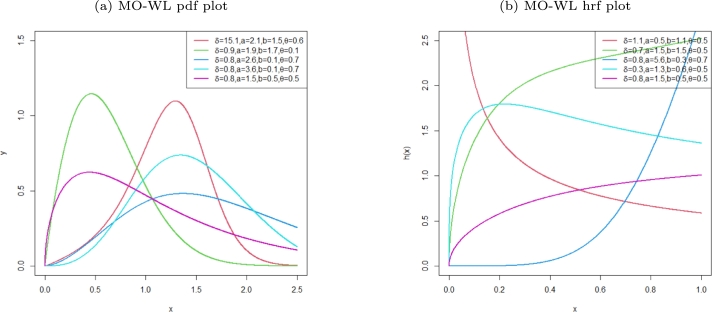
The pdf and hrf plots for the MO-WL distribution.

Fig. 1 shows plots associated with equation (4) and (5), it is clear that the model can handle symmetric data and right skewed datasets, and the hrf is exhibiting an decreasing, increasing and upside down bathtub shapes.

When a study is completed before all of the subjects have experienced the event of interest, censored data are produced. More details about censored data and the references it contains are discussed [12]. The application of the Marshall-Olkin Weibull Logarithmic (MO-WL) distribution, a specific case of the MO-GPS, to complete and censored data was the main focus of this work. The objectives of this essay are as follows:

• To presenting the distribution of the Marshall-Olkin Weibull Logarithmic (MO-WL). The model can be used to model different types of datasets because it contains numerous density shapes and hrf shapes.
• To apply the maximum likelihood estimation (MLE) technique to the estimation of the model parameters.
• To analyze real complete and type 1 censored datasets from different fields.

Here is the outline of the article: In Section 2, we estimate the parameters of the new model using the method of MLE for both censored and complete data sets. A Monte Carlo simulation study is presented in Section 3. Section 4 demonstrates the utility of the MO-WL distribution using three data sets. Finally, Section 5 offers concluding remarks.

## 2 Maximum likelihood estimation

In this section, we present the estimates of the unknown parameters of the MO-WL model for complete and censored data. We respectively discuss parameter estimation for complete and censored data. Let $x_{1},x_{2},...,x_{n}$ be a random sample of size *n* from the MO-WL distribution with pdf given in equation (4).

### 2.1 Estimation of parameters for uncensored data

Let $\Delta ={\left(\delta ,\theta ,a,b\right)}^{T}$ be the vector of model parameters. The log-likelihood function $\ell_{n}(\Delta )=\ell_{n}$ based on a random sample of size *n* from the MO-WL distribution is given by

(6) $$\ell_{n}=n\log(\theta \delta )+\sum_{i=1}^{n}\log(abx^{a-1}e^{-bx^{a}})-2\sum_{i=1}^{n}\log(1-\nabla )+\sum_{i=1}^{n}\log\left[{\left(1-\frac{\theta ℧}{1-\nabla }\right)}^{-1}\right]-n\log(-\log(1-\theta )),$$

where $\nabla =\overset{¯}{\delta }e^{-bx^{a}}$ and $℧=\delta e^{-bx^{a}}$. The partial derivatives of the log-likelihood function specified in equation (6) with respect to Δ must be executed, set to zero, and Δ must be solved for in order to obtain the likelihood estimates. Appendix A contains the formulae for the partial derivatives of the log-likelihood, as described in equation (6). These partial derivatives are clearly nonlinear in Δ. Consequently, in order to solve them, iterative techniques like the Newton-Raphson method are required [13], [14], [15], [16] and [17].

### 2.2 Estimation of parameters for censored data

Let the lifetime of the first *r* failed items be $x_{1},x_{2},...,x_{r}$, the censoring be $x_{r+1},x_{r+2},...,x_{n}$ and $\Delta ={\left[\delta ,\theta ,a,b\right]}^{T}$ be the vector of parameters. Following [12], the likelihood function of the censored data under the MO-WL model is given by

(7) $$\ell_{n}=\log(A)+n\log(\theta \delta )+\sum_{i=1}^{r}\log(abx^{a-1}e^{-bx^{a}})-2\sum_{i=1}^{r}\log(1-\nabla )+\sum_{i=1}^{r}\log\left[{\left(1-\frac{\theta ℧}{1-\nabla }\right)}^{-1}\right]-n\log(-\log(1-\theta ))+\sum_{i=r+1}^{n}\log\left[-\log\left(1-\frac{\theta ℧}{1-\nabla }\right)\right].$$

Comparably, for each parameter (Δ), we must take into account partial derivatives of the log-likelihood function specified in equation (7); we then set these derivatives to zero and iteratively solve for Δ. In Appendix A, the expressions for these partial derivatives are given.

## 3 Simulation study

To illustrate the convergence of the MLEs, we ran a simulation in this section using the R software (stats4) [14]. We used the MO-WL model to simulate 1000 samples iteratively, with n=25,50,100,200, and 400 sizes. The MLEs for each of the 1000 replications were found. The average bias (ABias) and the root mean square error (RMSE) were computed for the estimated parameter, $\overset{ˆ}{\kappa }$.

$$ABias(\overset{ˆ}{\kappa })=\frac{\sum_{i=1}^{1000}{\overset{ˆ}{\kappa }}_{i}}{1000}-\kappa ,\;\text{and}\;RMSE(\overset{ˆ}{\kappa })=\sqrt{\frac{\sum_{i=1}^{1000}{\left({\overset{ˆ}{\kappa }}_{i}-\kappa \right)}^{2}}{1000}},$$

respectively. Table 1, Table 2 show the results of the simulation for different values of δ,θ,a, and *b*. It is clear that the ABIAS and the RMSE decrease with increasing sample size *n*, showing the MLE's convergence. The simulation results show that when estimating parameters for the MO-WL model, maximum likelihood estimation produces accurate results.

**Table 1:** MO-WL distribution Simulated Results 1.

		(0.5,0.5,2.5,1.0)			(0.5,0.02,0.8,1.0)		
Parameter	Sample Size	MLE	RMSE	ABias	MLE	RMSE	A.Bias
*δ*	25	1.2072	1.7670	0.7072	0.7498	1.0450	0.2498
	50	1.3134	1.7907	0.8134	0.6390	0.7342	0.1390
	100	1.1786	1.2113	0.6786	0.6040	0.5438	0.1040
	200	0.9874	0.9488	0.4874	0.5898	0.4013	0.0898
	400	0.8081	0.5831	0.3081	0.5831	0.3561	0.0831

*θ*	25	0.6692	0.4054	0.1692	0.3911	0.5643	0.3711
	50	0.6804	0.4089	0.1804	0.2847	0.4641	0.2647
	100	0.7040	0.3964	0.2040	0.2355	0.4246	0.2155
	200	0.6689	0.3814	0.1689	0.1936	0.3756	0.1736
	400	0.6376	0.3686	0.1376	0.1769	0.3601	0.1569

*a*	25	2.9784	0.8097	0.4784	0.9596	0.2618	0.1596
	50	2.9112	0.6973	0.4112	0.9215	0.1902	0.1215
	100	2.7748	0.5085	0.2748	0.8821	0.1450	0.0821
	200	2.7083	0.4159	0.2083	0.8440	0.1022	0.0440
	400	2.6589	0.2951	0.1589	0.8368	0.0829	0.0368

*b*	25	0.9856	0.5902	-0.0144	0.8393	0.4301	-0.1607
	50	1.0203	0.5476	0.0203	0.8203	0.3881	-0.1797
	100	1.0250	0.4705	0.0250	0.8638	0.3427	-0.1362
	200	1.0087	0.4075	0.0087	0.9063	0.2750	-0.0937
	400	0.9757	0.2730	-0.0243	0.9197	0.2176	-0.0803

**Table 2:** MO-WL distribution Simulated Results 2.

		(0.5,0.5,1.8,1.0)			(0.5,0.5,1.4,1.0)		
Parameter	Sample Size	MLE	RMSE	Bias	MLE	RMSE	A.Bias
*δ*	25	1.1207	1.5045	0.6207	1.1635	1.7627	0.6635
	50	1.3014	1.7150	0.8014	1.1661	1.4356	0.6661
	100	1.1192	1.1835	0.6192	1.1948	1.4551	0.6948
	200	0.9354	0.8618	0.4354	0.9583	0.8890	0.4583
	400	0.7948	0.5612	0.2948	0.8130	0.5803	0.3130

*θ*	25	0.6308	0.4142	0.1308	0.7049	0.4118	0.2049
	50	0.6880	0.4053	0.1880	0.6415	0.4007	0.1415
	100	0.6710	0.3931	0.1710	0.6703	0.3990	0.1703
	200	0.6624	0.3855	0.1624	0.6610	0.3823	0.1610
	400	0.6324	0.3706	0.1324	0.6542	0.3719	0.1542

*a*	25	2.1994	0.5973	0.3994	1.7354	0.5033	0.3354
	50	2.0915	0.5178	0.2915	1.6198	0.3688	0.2198
	100	1.9898	0.3645	0.1898	1.5577	0.2943	0.1577
	200	1.9649	0.3104	0.1649	1.5228	0.2475	0.1228
	400	1.9163	0.2131	0.1163	1.4976	0.1744	0.0976

*b*	25	0.9623	0.5856	-0.0377	0.9165	0.6223	-0.0835
	50	1.0194	0.5912	0.0194	0.9887	0.5444	-0.0113
	100	1.0261	0.4863	0.0261	1.0173	0.4876	0.0173
	200	0.9742	0.3735	-0.0258	0.9906	0.4025	-0.0094
	400	0.9741	0.2689	-0.0259	0.9676	0.2772	-0.0324

## 4 Applications

**Figure 2:**
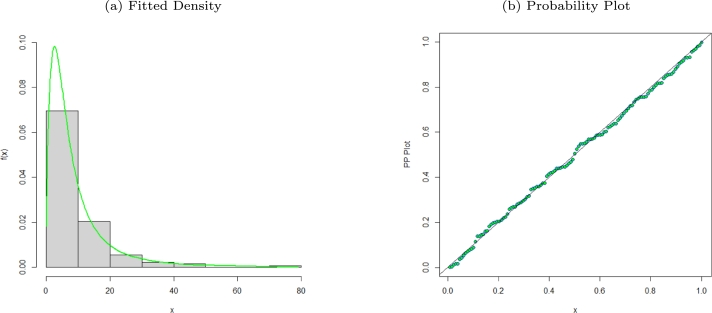
Fitted density and probability plot of the MO-WL distribution for the remission times of 128 bladder cancer patients data.

**Figure 3:**
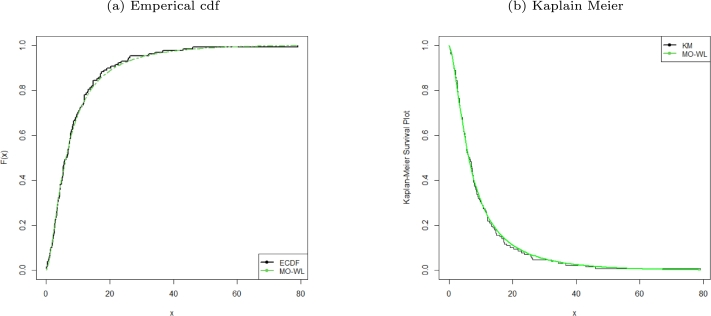
ECDF and KM survival plots of the MO-WL distribution for the remission times of 128 bladder cancer patients data.

**Figure 4:**
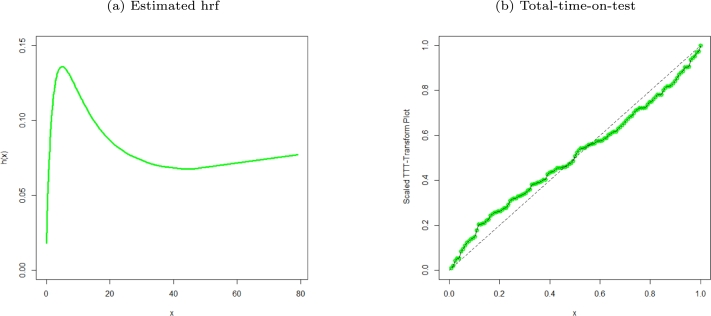
Estimated hrf plot and TTT of the MO-WL distribution for the remission times of 128 bladder cancer patients data.

**Figure 5:**
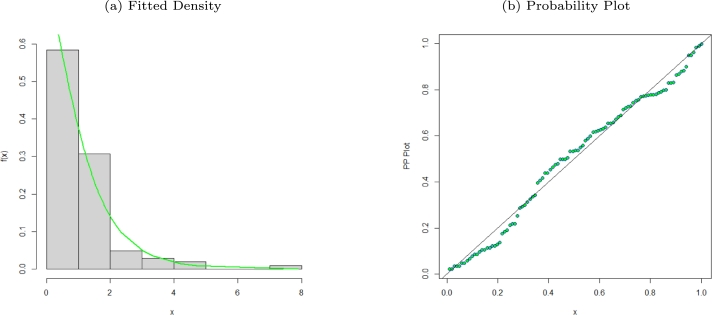
Fitted density and probability plot of the MO-WL distribution for the Kevlar data.

**Figure 6:**
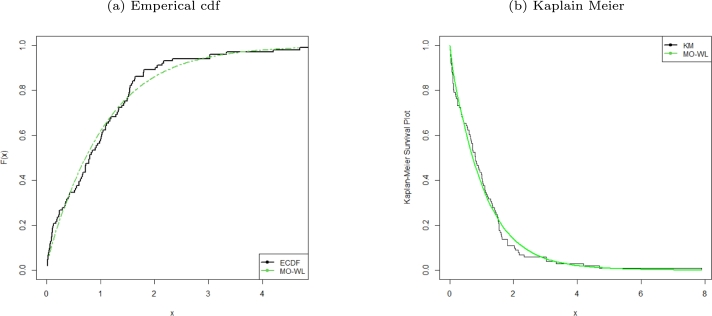
ECDF and KM survival plots of the MO-WL distribution for the Kevlar data.

**Figure 7:**
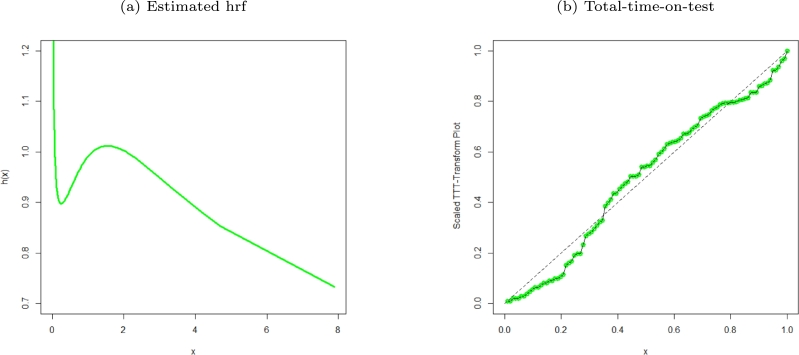
Estimated hrf plot and TTT of the MO-WL distribution for the Kevlar data.

**Figure 8:**
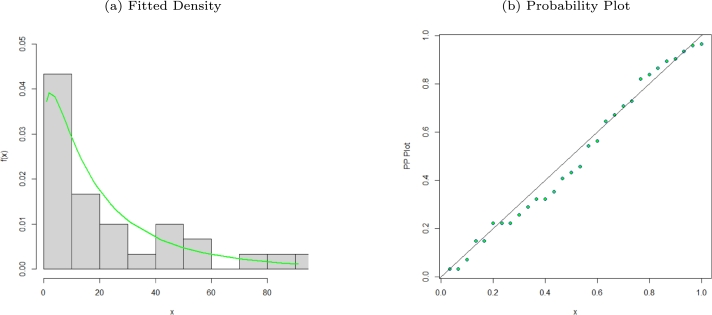
Fitted density and probability plot of the MO-WL distribution for the censored data.

**Figure 9:**
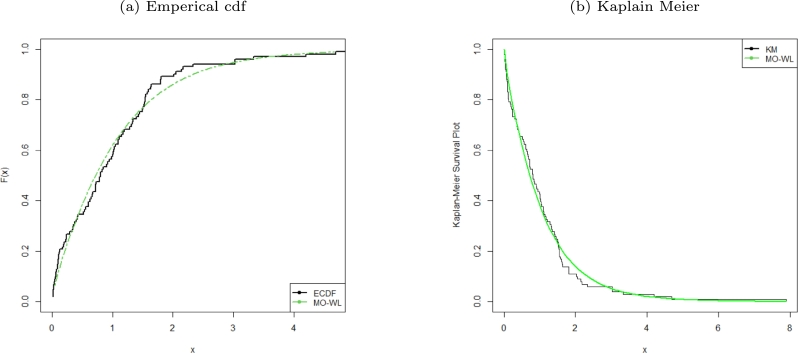
ECDF and KM survival plots of the MO-WL distribution for the censored data.

**Figure 10:**
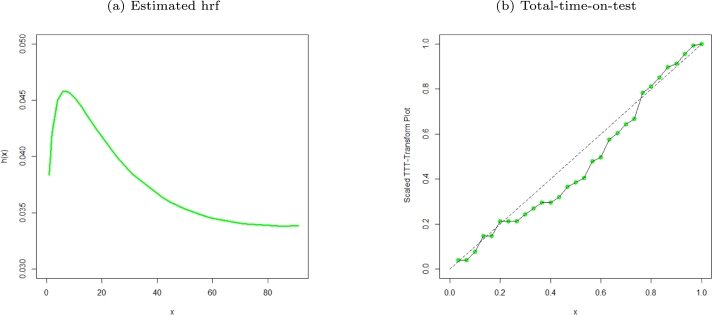
Estimated hrf plot and TTT of the MO-WL distribution for the censored data.

We demonstrate the usefulness of the MO-WL distribution with an actual data set. The MO-WL and non-nested comparative models' goodness-of-fit (GoF) statistics are shown. The GoF statistics −2loglikelihood(−2logL), the Bayesian Information Criterion (BIC), the Consistent Akaike Information Criterion (AICC), the Cramer-Von Mises $\left(W^{⁎}\right)$, and the Andersen Darling $\left(A^{⁎}\right)$, as stated by [18], were used to evaluate the performance of the model. The Kolmogorov-Smirnov (K-S) GoF measure was also calculated by us. The best-fitting model is the one that has the lowest values for these statistics and the highest p-value for the K-S statistic. Using the **nlm** function, we estimated the model parameters using R software. Model parameter estimates with standard errors in parentheses are given in Table 3, Table 5, Table 7 with the GoF statistics for the selected data sets presented in Table 4, Table 6, Table 8. Figure 2, Figure 3, Figure 4, Figure 5, Figure 6, Figure 7, Figure 8, Figure 9, Figure 10 also present plots of the fitted densities, the histogram of the data, probability plots, Kaplain Meier (KM), empirical cumulative distribution function (ECDF) and total-time-on-test (TTT) plots [19] to show how well our model fits the observed data sets.

**Table 3:** Parameter estimates for various models fitted for the remission times of 128 bladder cancer patients data set.

Estimates				
Model	*δ*	*θ*	a	b
MO-WL	0.0638	6.8469×10^−6^	1.6042	0.0033
	(0.0642)	(0.5025)	(0.1629)	(0.0038)
	*α*	*λ*	*γ*	*θ*
OW-TL-LLoGL	0.1801	0.9741	0.9771	4.59×10^−9^
	(0.0305)	(0.1372)	(0.2614)	(0.0069)
	*p*	*α*	*β*	*λ*
EGEL	1.4532×10^−7^	0.0870	1.2180	1.3912
	(0.0391)	(0.0097)	(0.1490)	(0.0006)
	*θ*	*α*	*β*	*ω*
EPLP	2.2382×10^−7^	0.5666	0.8182	2.7647
	(0.0305)	(0.1016)	(0.3112)	(1.2868)
	*c*	*s*	*α*	*λ*
ELLoGP	0.7517	1.0984	1.1102	3.0195×10^−9^
	(0.0721)	(0.1809)	(0.1021)	(0.0051)
	*β*	*λ*	*θ*	*γ*
TLGW	0.0235	4.5143	5.7081	0.0913
	(0.0167)	(1.1940)	(1.8968)	(0.0296)
	*β*	*λ*	*θ*	*γ*
GGP	1.1012e	0.5063	0.2062	2.2494×10^−10^
	(0.6681)	(0.0575)	(0.0600)	(0.0072)

**Table 4:** GoF statistics for various models fitted for the remission times of 128 bladder cancer patients data set.

GoF Statistics								
Model	−2log⁡L	*AIC*	*AICC*	*BIC*	*W*^⁎^	*A*^⁎^	K-S	p-value
MO-WL	820.1842	828.1842	828.5094	839.5923	0.0284	0.2041	0.0324	0.9993
OW-TL-LLoGL	1118.988	1126.988	1127.314	1138.396	5.0262	24.9088	0.9166	2.2×10^−16^
EGEL	826.1552	834.1552	834.4804	845.5633	0.1122	0.6741	0.0725	0.5111
EPLP	820.867	828.867	829.1922	840.2751	0.0392	0.2568	0.0429	0.9725
ELLoGP	982.851	990.8544	991.1796	1002.263	0.1863	1.2345	0.4709	2.2×10^−16^
TLGW	821.1583	829.1583	829.4836	840.5665	0.0417	0.2747	0.0438	0.9662
GGP	934.7457	942.7488	943.074	954.1569	0.2243	1.3329	0.3406	2.518×10^−13^

We compared the MO-WL distribution with other competing four parameter non-nested models, namely the Exponentiated Generalized Logarithmic (EGEL) distribution [16], Odd Weibull-Topp-Leone-Log Logistic Logarithmic (OW-TL-LLoGL) distribution [20], Exponentiated Power Lindley Poisson (EPLP) distribution [21], Exponentiated Log-Logistic Poisson (ELLoGP) [22], Topp-Leone- Gompertz-Weibull (TLGW) [23] and Generalized Gompertz-Poisson (GGP) [24].

### 4.1 Complete data

#### 4.1.1 Remission times of 128 bladder cancer patients

The data consists of the remission times of 128 bladder cancer patients (see [25] for details). Table 3 shows the MLEs parameters with standard errors in parentheses. The estimated variance-covariance matrix is provided in Appendix B. Consequently, the estimated 95% confidence intervals for the model parameters are given by δ∈[0.0638±0.1259], $\theta \in [6.8469\times 10^{-6}\pm 0.9850]$, a∈[1.6042±0.3193] and b∈[0.0033±0.0076]. Table 4 presents the GoF statistics, and as shown by the lowest goodness-of-fit statistics and the highest K-S P-value, we conclude that the proposed model provides the best overall fit to the remission times data set. It is evident from Figure 2, Figure 3, Figure 4 that the MO-WL distribution offers more flexibility and better fit for the data set of 128 bladder cancer patients' remission times. The histogram demonstrates that the highly tailed data set can be accommodated by the MO-WL distribution. Our model exhibits an excellent fit by imitating the probability line. Additionally, we note that the ECDF and fitted KM curves are near the MO-WL, indicating improved model performance. Moreover, the fitted hrf displays the shape of an upside-down bathtub, which matches the TTT plot.

#### 4.1.2 Kevlar 49/epoxy strands

We have comprehensive data with precise periods of failure since the data represents the stress-rupture life of Kevlar 49/epoxy strands that were continuously subjected to sustained pressure at a 90 stress level until all had failed. The data was also used in [26], [27], [28] and [29]. Table 5 shows the parameter values and standard errors in parentheses, and Appendix B shows the estimated variance-covariance matrix for the MO-WL model on Kevlar 49/epoxy strands data. The estimated 95% confidence intervals for the MO-WL model parameters are given by δ∈[3.9166±7.5644], $\theta \in [8.2214\times 10^{-5}\pm 0.1502]$, a∈[0.6931±0.3332] and b∈[1.9955±1.6432]. Table 6, presents the goodness-of-fit measures, and according to the findings, we note that the MO-WL has the highest p-value and the lowest GoF statistics when compared to the competing models. As a result, we conclude that the MO-WL model outperforms the competing models. Figure 5, Figure 6, Figure 7 display clearly that the MO-WL distribution provides better fit and flexibility in fitting the Kevlar 49/epoxy strands data set. The histogram demonstrates that the highly tailed data set can be accommodated by the MO-WL distribution. Our model exhibits an excellent fit by imitating the probability line. Additionally, we see that the fitted ECDF and KM curves closely resemble the MO-WL curve, indicating improved model performance. Additionally, as can be seen from the TTT plot, the fitted hrf displays a bathtub form followed by an upside-down bathtub shape that is comparable to the dataset.

**Table 5:** Parameter estimates with standard errors in parentheses for various models fitted for the stress-rupture life of Kevlar 49/epoxy strands data set.

Estimates				
Model	*δ*	*θ*	a	b
MO-WL	3.9166	8.2214×10^−5^	0.6931	1.9955
	(3.8594)	(0.0766)	(0.1700)	(0.8383)
	*α*	*λ*	*γ*	*θ*
OW-TL-LLoGL	0.4286	1.2941	1.3794	1.1844×10^−8^
	(0.1199)	(0.3750)	(0.2288)	(0.0154)
	*p*	*α*	*β*	*λ*
EGEL	3.7308×10^−8^	0.6013	0.8575	1.3988
	(0.0237)	(0.2142)	(0.1106)	(0.4982)
	*θ*	*α*	*β*	*ω*
EPLP	1.1683	0.7894	1.7951	0.9385
	(1.2586)	(0.2025)	(0.6111)	(0.3828)
	*c*	*s*	*α*	*λ*
EBXIIP	0.7150	0.2935	1.0195	2.6980×10^−9^
	(0.0682)	(0.0661)	(0.1232)	(0.0059)
	*β*	*λ*	*θ*	*γ*
TLGW	0.3735	1.5051	2.4139	0.3145
	(0.4298)	(2.0039)	(0.9797)	(0.2598)
	*β*	*λ*	*θ*	*γ*
GGP	1.0515	0.6645	0.9400	5.1908×10^−8^
	(1.3346)	(0.2262)	(0.1716)	(0.01848)

**Table 6:** GoF statistics for various models fitted for the stress-rupture life of Kevlar 49/epoxy strands data set.

GoF Statistics								
Model	−2log⁡L	*AIC*	*AICC*	*BIC*	*W*^⁎^	*A*^⁎^	K-S	p-value
MO-WL	203.6843	211.6843	212.101	222.1448	0.1189	0.7368	0.0704	0.6985
OW-TL-LLoGL	218.9	226.9	227.4	237.4	0.2593	2.2359	0.3024	1.892×10^−8^
EGEL	205.8	213.8	214.3	224.3	0.1815	1.0312	0.0815	0.5129
EPLP	204.4	212.4	212.9	222.9	0.1348	0.8140	0.0708	0.6921
EBXIIP	271.0	279.0	279.5	289.5	0.6774	3.6501	0.2483	7.7780×10^−6^
TLGW	205.9	213.9	214.3	224.4	0.1681	0.9760	0.0842	0.4706
GGP	205.3	213.3	213.7	223.8	0.1237	0.7737	0.0828	0.4933

### 4.2 Censored dataset

#### 4.2.1 Leukemia patients dataset

The censored data of consideration represent the times of remission, in weeks, for a group of 30 leukemia patients who underwent similar treatment (see [30], pp. 139). Table 7 shows the MLEs parameters together with corresponding standard errors in parentheses. The estimated values for the MO-WL variance-covariance matrix are provided in Appendix B. Consequently, the 95% confidence intervals (CIs) for the novel model parameters are given by δ∈[0.3812±0.9488], $\theta \in [3.8855\times 10^{-5}\pm 0.3087]$, a∈[1.1699±0.5634] and b∈[0.0127±0.0431]. For the chosen models, the GoF statistics are displayed in Table 8. We found that the MO-WL distribution has the overall best fit on the censored data set and has the highest p-value with the lowest GoF statistic values. We get the conclusion that the MO-WL performs superior than the rival non-nested models shown in the table. It can be shown from Figure 8, Figure 9, Figure 10 that the MO-WL distribution fits this censored data set more flexible and well. The histogram demonstrates that the censored data set can be accommodated by the MO-WL distribution. Our model exhibits an excellent fit by imitating the probability line. Additionally, we note that the fitted ECDF and KM curves closely resemble our model, demonstrating the improved performance of our model. Also, the TTT plot confirms that the fitted hrf has the shape of an upside-down bathtub.

**Table 7:** Parameter estimates with standard errors in parentheses for various models fitted for the censored data set.

Estimates				
Model	*δ*	*θ*	a	b
MO-WL	0.3812	3.8855×10^−5^	1.1699	0.0127
	(0.4841)	(0.2874)	(0.2874)	(0.0220)
	*p*	*α*	*β*	*λ*
EGEL	7.0818×10^−9^	0.0326	1.5154	1.5746
	(0.0160)	(0.1588)	(0.4153)	(7.6617)
	*c*	*s*	*α*	*λ*
EBXIIP	0.7067	0.8829	0.7603	7.8400×10^−10^
	(0.1508)	(0.3027)	(0.1399)	(0.0054)
	*β*	*λ*	*θ*	*γ*
TLGW	0.1760	0.0986	2.9278	0.4662
	(0.4126)	(0.4939)	(5.6767)	(0.6266)
	*β*	*λ*	*θ*	*γ*
GGP	1.1001	0.5007	0.2223	1.2093×10^−11^
	(0.6541)	(0.0963)	(0.0282)	(8.0252×10^−4^)

**Table 8:** Parameter estimates and GoF statistics for various models fitted for the censored data set.

GoF Statistics								
Model	−2log⁡L	*AIC*	*AICC*	*BIC*	*W*^⁎^	*A*^⁎^	K-S	p-value
MO-WL	253.3391	261.3391	262.9391	266.9439	0.0382	0.2530	0.0877	0.9751
EGEL	257.6304	265.6303	267.2303	271.2351	0.0522	0.3203	0.1821	0.2729
EBXIIP	331.4113	339.4113	341.0113	345.0161	0.0421	0.39336	0.6987	3.7770×10^−13^
TLGW	253.4068	261.4068	263.0068	267.0116	0.0396	0.2600	0.0857	0.9082
GGP	438.8484	446.8484	448.4484	452.4532	0.0953	0.5619	0.6167	2.459×10^−10^

## 5 Conclusion

We presented a distribution called Marshall-Olkin Weibull Logarithmic. To demonstrate the value and applicability of the model, applications of the MO-WL under complete and censored real data sets are provided. It is evident from Table 4, Table 6, Table 8 that the MO-WL offers the best overall fit and has the highest P-value with the lowest goodness-of-fit statistic values; as a result, the distribution fits the datasets superior than the non-nested models presented in this paper. Thus the MO-WL distribution could play a reasonable role as a candidate for modeling real-life datasets. Although the distribution performed well in modeling presented complete and type I censored datasets, more research should be carried on the distribution fitting different censoring schemes like type I and type II double censoring schemes.

## Declarations

### Funding statement

This research did not receive any specific grant from funding agencies in the public, commercial, or not-for-profit sectors.

## CRediT authorship contribution statement

**Regent Retrospect Musekwa:** Writing – review & editing, Writing – original draft, Visualization, Software, Project administration, Methodology, Investigation, Formal analysis, Data curation, Conceptualization. **Lesego Gabaitiri:** Writing – review & editing, Supervision, Resources, Conceptualization. **Boikanyo Makubate:** Writing – review & editing, Writing – original draft, Supervision, Software, Resources, Project administration, Methodology, Investigation, Formal analysis, Conceptualization.

## Declaration of Competing Interest

The authors declare that they have no known competing financial interests or personal relationships that could have appeared to influence the work reported in this paper.

## Data Availability

Data included in this article is referenced in the article.

## Appendix A Estimation of parameters for uncensored and censored data

### A.1 Estimation of parameters for complete data

$$\frac{\partial \ell_{n}}{\partial \theta }=\frac{n}{\theta }+\frac{n{\left(1-\theta \right)}^{-1}}{\log(1-\theta )}+\sum_{i=1}^{n}{\left(1-\frac{\theta ℧}{1-\nabla }\right)}^{-1}\left(\frac{℧}{1-\nabla }\right)\frac{\partial \ell_{n}}{\partial \delta }=\frac{n}{\delta }-2\sum_{i=1}^{n}\frac{e^{-bx^{a}}}{1-\nabla }+\sum_{i=1}^{n}{\left(1-\frac{\theta ℧}{1-\nabla }\right)}^{-1}\times (\frac{\theta (1-e^{-bx^{a}})e^{-bx^{a}}}{\left(1-\nabla \right)}),\frac{\partial \ell_{n}}{\partial a}=\sum_{i=1}^{n}\frac{bx^{a-1}+a((a-1)\log(x)x^{2(a-1)})}{abx^{a-1}}-\sum_{i=1}^{n}bx^{a}\log(x)-2\sum_{i=1}^{n}\frac{\nabla b\log(x)x^{a}}{1-\nabla }+\sum_{i=1}^{n}\frac{1-\nabla -\theta ℧}{1-\nabla }\times [(1-\nabla -\theta ℧)(-e^{-bx^{a}}bx^{a}\log(a))-(1-\nabla )\times (\nabla bx^{a}\log(x)+\theta b℧x^{a}\log(x))]\times {\left(1-\nabla -\theta ℧\right)}^{-2},$$

and

$$\frac{\partial \ell_{n}}{\partial b}=\frac{n}{b}-\sum_{i=1}^{n}x^{a}-2\sum_{i=1}^{n}\frac{\nabla x^{a}}{1-\nabla }+\sum_{i=1}^{n}\frac{1-\nabla -\theta ℧}{1-\nabla }\times [(1-\nabla -\theta \delta ℧)(\nabla x^{a})-(1-\nabla )(\nabla x^{a}+\theta ℧x^{a})]\times {\left(1-\nabla -\theta ℧\right)}^{-2},$$

where $\nabla =\overset{¯}{\delta }e^{-bx^{a}}$ and $℧=\delta e^{-bx^{a}}$.

### A.2 Estimation of parameters for censored data

$$\frac{\partial \ell_{n}}{\partial \theta }=\frac{n}{\theta }+\frac{2n{\left(1-\theta \right)}^{-1}}{\log(1-\theta )}+\sum_{i=1}^{r}{\left(1-\frac{\theta ℧}{1-\nabla }\right)}^{-1}\left(\frac{℧}{1-\nabla }\right)-\sum_{i=r+1}^{n}\left(\frac{{\left(1-\frac{\theta ℧}{1-\nabla }\right)}^{-1}}{\log\left(1-\frac{\theta ℧}{1-\nabla }\right)}\left(\frac{℧}{1-\nabla }\right)\right),\frac{\partial \ell_{n}}{\partial \delta }=\frac{n}{\delta }-2\sum_{i=1}^{r}\frac{e^{-bx^{a}}}{1-\nabla }+\sum_{i=1}^{r}{\left(1-\frac{\theta ℧}{1-\nabla }\right)}^{-1}\times (\frac{\theta (1-e^{-bx^{a}})e^{-bx^{a}}}{\left(1-\nabla \right)})-\sum_{i=r+1}^{n}\frac{{\left(1-\frac{\theta ℧}{1-\nabla }\right)}^{-1}}{\log\left(1-\frac{\theta ℧}{1-\nabla }\right)}\times (\frac{\theta (1-e^{-bx^{a}})e^{-bx^{a}}}{\left(1-\nabla \right)}),\frac{\partial \ell_{n}}{\partial a}=\sum_{i=1}^{n}\frac{bx^{a-1}+a((a-1)\log(x)x^{2(a-1)})}{abx^{a-1}}-\sum_{i=1}^{n}bx^{a}\log(x)-2\sum_{i=1}^{n}\frac{\nabla b\log(x)x^{a}}{1-\nabla }+\sum_{i=1}^{n}\frac{1-\nabla -\theta ℧}{1-\nabla }\times [(1-\nabla )(\nabla bx^{a}\log(x)+\theta b℧x^{a}\log(x))-(1-\nabla -\theta ℧)(-e^{-bx^{a}}bx^{a}\log(a))]÷{\left(1-\nabla -\theta ℧\right)}^{2}+\sum_{i=r+1}^{n}{\left[\log\left(\frac{1-\nabla -\theta ℧}{1-\nabla }\right)\right]}^{-1}{\left(\frac{1-\nabla -\theta ℧}{1-\nabla }\right)}^{-1}\times [(1-\nabla -\theta ℧)(-e^{-bx^{a}}bx^{a}\log(a))-(1-\nabla )(\nabla bx^{a}\log(x)+\theta b℧x^{a}\log(x))]\times {\left(1-\nabla \right)}^{-2},$$

and

$$\frac{\partial \ell_{n}}{\partial b}=\frac{n}{b}-\sum_{i=1}^{n}x^{a}-2\sum_{i=1}^{n}\frac{\nabla x^{a}}{1-\nabla }+\sum_{i=1}^{n}\frac{1-\nabla -\theta ℧}{1-\nabla }\times [(1-\nabla -\theta ℧)(\nabla x^{a})-(1-\nabla )(\nabla x^{a}+\theta ℧x^{a})]÷{\left(1-\nabla -\theta ℧\right)}^{2}+\sum_{i=r+1}^{n}{\left[\log\left(\frac{1-\nabla -\theta ℧}{1-\nabla }\right)\right]}^{-1}{\left(\frac{1-\nabla -\theta ℧}{1-\nabla }\right)}^{-1}\times [(1-\nabla )(\nabla x^{a}+\theta ℧x^{a})-(1-\nabla -\theta ℧)(\nabla x^{a})]\times {\left(1-\nabla \right)}^{-2}$$

where $\nabla =\overset{¯}{\delta }e^{-bx^{a}}$ and $℧=\delta e^{-bx^{a}}$.

## Appendix B Variance-covariance matrices

The variance-covariance matrix for MO-WL model on the remission times of 128 bladder cancer patients data set is given by

$$\left[\begin{array}{cccc} 0.0041 & 0.0081 & -0.0078 & 2.3634\times 10^{-4}\\ 0.0081 & 0.2525 & 5.6918\times 10^{-8} & -3.1323\times 10^{-10}\\ -0.0078 & 5.6918\times 10^{-8} & 0.0265 & -5.5639\times 10^{-4}\\ 2.3634\times 10^{-4} & -3.1323\times 10^{-10} & -5.5639\times 10^{-4} & 1.5173\times 10^{-5} \end{array}\right].$$

The variance-covariance matrix for MO-WL model on epoxy strands failure at 90% stress level data set is given by

$$\left[\begin{array}{cccc} 14.8949 & 0.0115 & -0.613 & 53.1821\\ 0.0115 & 0.0058 & 5.6183\times 10^{-8} & -2.4730\times 10^{-7}\\ -0.6135 & 5.6183\times 10^{-8} & 0.0289 & -0.1345\\ 3.1821 & -2.4730\times 10^{-7} & -0.1345 & 0.7028 \end{array}\right].$$

The variance-covariance matrix for MO-WL model on the censored data set is given by

$$\left[\begin{array}{cccc} 0.2343 & -0.0047 & -0.1161 & 0.0100\\ -0.0047 & 2.4803\times 10^{-2} & -1.2529\times 10^{-6} & 1.0680\times 10^{-7}\\ -0.1161 & -1.2529\times 10^{-6} & 8.2651\times 10^{-2} & -6.0580\times 10^{-3}\\ 0.0100 & 1.0680\times 10^{-7} & -6.0580\times 10^{-3} & 4.8411\times 10^{-4} \end{array}\right].$$
